# Ferroptosis-Like Death Induction in *Saccharomyces cerevisiae* by Gold Nanoparticles

**DOI:** 10.4014/jmb.2501.01029

**Published:** 2025-04-27

**Authors:** Min Seok Kwun, Dong Gun Lee

**Affiliations:** 1 School of Life Science, BK21 FOUR KNU Creative BioResearch Group, Kyungpook National University, Daegu 41566, Republic of Korea; 2 Institute of Life Science and Biotechnology, Kyungpook National University, Daegu 41566, Republic of Korea

**Keywords:** Ferroptosis-like death, gold nanoparticle, *Saccharomyces cerevisiae*, lipid peroxidation

## Abstract

Ferroptosis, a novel form of regulated cell death (RCD), has emerged as a promising therapeutic strategy for cancer treatment. While gold nanoparticles (AuNPs) are known to induce cell death and ferroptosis in combination with certain antibiotics, the mechanisms underlying ferroptosis in microorganisms remain poorly understood. This study aimed to investigate whether AuNPs induce ferroptosis-like cell death in the eukaryotic microbe *Saccharomyces cerevisiae*. Our findings revealed that AuNPs significantly reduced cell viability in *S. cerevisiae*, suggesting their ability to trigger cell death. Ferroptosis-related precursors, including intracellular iron overload and depletion of glutathione (GSH), were observed, leading to the inactivation of glutathione peroxidase (GPx). These changes were associated with the accumulation of reactive oxygen species (ROS) and lipid peroxidation, which amplified oxidative stress within the cells. Elevated ROS levels and lipid peroxidation further resulted in membrane rupture and the formation of 8-hydroxydeoxyguanosine, indicating DNA damage. Mitochondrial dysfunction, a hallmark of ferroptosis, was also evident. AuNP treatment caused mitochondrial membrane potential hyperpolarization and a reduction in mitochondrial membrane density. Unlike previously characterized forms of RCD, ferroptosis-like death in *S. cerevisiae* did not involve chromatin condensation, DNA fragmentation, or metacaspase activation. Finally, ferroptosis-like characteristics were confirmed using Liperfluo, a lipid ROS-specific probe. In conclusion, this study demonstrated that AuNPs can induce ferroptosis-like cell death in *S. cerevisiae*. These findings highlight the potential of AuNPs as antifungal agents and contribute to the broader understanding of ferroptosis in eukaryotic microbes.

## Introduction

Nanoparticles (NPs) are ultrafine particles that are 1-100 nm. Because of their physical and chemical properties resulting from their small size, they are welcomed as medical or biological application [1, 2]. Most NPs are considered suitable for drug delivery and treatment because they are the proper size to interface biomolecules, such as DNA and proteins [3-5]. In particular, their high surface-to-volume ratio allows for enhanced interaction with cellular components, improving drug loading efficiency and targeted delivery [6]. Additionally, their tunable surface chemistry enables functionalization with ligands, antibodies, or therapeutic agents, making them highly adaptable for biomedical applications. Various types of NPs, including metal, polymeric, and lipid-based nanoparticles, have been developed to optimize biocompatibility and therapeutic potential [7]. Among metallic nanoparticles, gold nanoparticles (AuNPs) and silver nanoparticles (AgNPs) have attracted significant attention. AuNPs are employed in various biomedical fields, such as diagnosis, cancer treatment, and nanobiotechnology based on their low toxicity and ability to regulate membrane penetration [8, 9]. For example, in mammalian cancer cells, AuNPs are known to significantly reduce cell viability, which not only inhibit cell proliferation, but also cause cell-cycle arrest [10, 11]. Additionally, this nanomaterial is also regarded as a potential anticancer agent because it induces cell death, such as apoptosis and necrosis depending on time and concentration [12, 13]. Furthermore, several papers have reported that AuNPs form a complex with antibiotics to exhibit synergistic effects or enhance the antimicrobial effect while acting as antibiotic carriers [14-16]. Many studies have found that AuNPs exhibit activity in bacteria, which are mechanisms to repress protein activity and weaken DNA replication. However, mechanisms for anti-yeast or anti-fungal activity require further exploration [17, 18].

Recently, a novel form of regulated cell death (RCD) distinct from apoptosis and autophagy has been identified. This process, called "ferroptosis," was first described by Dixon *et al*. in 2012 [19]. Unlike previously known RCD mechanisms, ferroptosis exhibits unique morphological features, including the disappearance of mitochondrial cristae, a reduction in mitochondrial volume, and the breakdown of the outer mitochondrial membrane [20, 21]. Additionally, ferroptosis is characterized by biochemical changes such as excessive intracellular iron accumulation and the oxidation of polyunsaturated fatty acids (PUFAs) [22, 23]. The underlying mechanism of ferroptosis is categorized into two primary pathways. The first involves the inactivation of glutathione peroxidase 4 (GPx4) due to a deficiency in glutathione (GSH), which arises from a lack of cysteine, a precursor of GSH [24]. Since GPx4 is an endogenous enzyme that prevents lipid peroxidation, its dysfunction leads to ferroptosis by failing to maintain the GSH-dependent antioxidant system [25]. The second pathway is associated with the accumulation of iron within cells [26]. Iron plays a key role in the generation of reactive oxygen species (ROS) and acts as a cofactor for lipoxygenase, facilitating PUFA oxidation [20, 27]. When intracellular iron levels become excessive, ROS production increases, and PUFA oxidation contributes to the induction of ferroptosis. Various inducers of ferroptosis have been identified based on these mechanisms. For instance, erastin and sulfasalazine inhibit cysteine uptake, leading to GSH depletion, while RSL-3 directly inactivates GPx4 [28, 29].

Lately, some papers have been published that NPs can mediate ferroptosis, and ferroptosis is welcomed as a new therapeutic target for various diseases. [30-34]. However, while the effects and mechanisms of AuNPs in mammalian cells are well known, research on the relationship between eukaryotic microbe and AuNPs is poor. Therefore, our study focuses on using *S. cerevisiae*, which has a homologous enzyme similar to mammalian cells, as a model organism to manifest whether AuNPs exhibit ferroptosis-like responses in *S. cerevisiae* [35, 36].

## Materials and Methods

### Preparation of Strain, Compound, and Cell Culture Conditions

*Saccharomyces cerevisiae* (KCTC 7296) was obtained from the Korean Collection for Type Cultures (KCTC, Republic of Korea). Gold nanoparticles (AuNPs, Sigma-Aldrich, USA) were 30 nm in diameter and diluted in phosphate-buffered saline (PBS) at a concentration of 50 μg/ml. The typical ferroptosis-inducing agent, erastin (10 μg/ml, Sigma), was used as a positive control and dissolved in dimethyl sulfoxide (DMSO). *S. cerevisiae* cells were grown in YPD (yeast extract-peptone-dextrose) broth (BD Bioscience, USA) and inoculated into YPD broth for all experiments under aerobic conditions at 30°C and 120 rpm. Yeast strain in the exponential phase was harvested and then resuspended in PBS.

### Cell Viability Assay

3-(4,5-dimethylthiazol-2-yl)-2,5-diphenyltetrazolium bromide (MTT, Sigma) assay was applied to test cell viability [37]. First, 5, 10, 20, and 40 μg/ml of AuNPs and 5, 10, 20, 40 μg/ml of erastin were treated in *S. cerevisiae*, respectively. After 2 h incubation at 30°C, the cells were centrifuged, and YPD medium was eliminated. Pellets were suspended with PBS, and 5 mg/ml MTT solution was allocated. Following incubation, the cells were centrifuged at 12,000 rpm, and the supernatant was eliminated. Next, 400 μl DMSO was allotted to each sample until the pellet was completely mixed. A fused sample was added to 96-well plates, and cell viability was determined at 580 nm absorbance using a microtiter ELISA reader (BioTek Instruments, USA).

### Measurement of Intracellular Iron Overload

An iron assay kit (Sigma) was used to monitor the iron accumulation in *S. cerevisiae*. This kit directly measures total iron levels in cells. Briefly, cells were treated with AuNPs or erastin and incubated for 2 h at 30°C. First, the cells were centrifuged at 12,000 rpm for 5 min and then iron assay buffer was added up to 10 times the volume of the pellets to each sample. Cell lysis was performed using the bead-beating method with 1 mm glass beads (Glastechnique). The disruption process involved four cycles of 1.5 min bead vortexing followed by 1 min incubation on ice. After disruption, the samples were centrifuged at 12,000 rpm for 10 min. The supernatant from each sample (50 μl) was transferred to a 96-well plate, and iron assay buffer (50 μl) was added to make the total volume 100 μl. Then, 5 μl of iron reducer was added and incubated for 30 min. After incubation, 100 μl of iron probe was treated and incubated for 1 h. Finally, the absorbance was measured at 600 nm, and the total iron levels were calculated according to standard curves (created based on the manufacturer's recommendations, Sigma).

### Measurement of Intracellular Glutathione (GSH) Levels

GSH is a peptide consisting of glutamic acid, cysteine and glycine and is present in almost all eukaryotes and some prokaryotes [38-40]. To measure the quantitative GSH concentration, already described protocols had been modified to apply to yeast cells [41-43]. First, *S. cerevisiae* cells were incubated with AuNPs or erastin for 2 h at 30°C. After incubation and centrifugation, the cells were suspended in 5% 5-sulphosalicylic acid (SSA, Sigma) and lysed by repeating freeze-thaw cycles. Before proceeding to the next step, the total protein level was assessed according to the Bradford method, and the protein level of each treated sample was calculated [44, 45]. Next, 100 μl of 0.01% dithionitrobenzoate (Sigma) was added to each sample for 15 min, and lastly, the absorbance was measured at 415 nm using a microtiter ELISA reader.

### Detection of Glutathione Peroxidase (GPx) Activity

GPx activity was assessed following a previously established method with slight modifications. The activity was determined by measuring the coupled reaction with glutathione reductase (GR). A reaction mixture consisting of 350 μl yeast cell extract, 100 μl GR (2.5 U/ml in phosphate buffer), and 100 μl reduced GSH was prepared in a 1 ml cuvette. After a 5-min incubation at 30°C, 100 μl NADPH was added, and the baseline oxidation of NADPH, independent of AuNPs or erastin, was monitored for 3 min. The reaction was then initiated by the addition of AuNPs or erastin, and the subsequent NADPH oxidation was measured at 340 nm. To account for nonenzymatic reduction caused by AuNPs or erastin, the cell extract was replaced with phosphate buffer as a control. GPx activity was calculated based on the reduction in NADPH absorbance, measured using a spectrophotometer (DU530, Beckman, USA).

### Detection of ROS Generation

Although many other molecules are included in ROS, H_2_O_2_, O_2_^-^ and OH∙ are considered as the typical ROS [46, 47]. Dihydroethidium (DHE, Sigma) was used to detect H_2_O_2_ or O_2_^-^ generation, and 3'-(p-hydroxyphenyl)(HPF, Molecular Probes, USA) was used to detect OH∙ generation [48-50]. Furthermore, the lipid ROS scavenger and ferroptosis inhibitor, ferrostatin-1 (10 mg/ml, Sigma), was dissolved in DMSO [51, 52]. These scavengers were pre-incubated for 5 min before AuNPs were treated. Next, *S. cerevisiae* cells were incubated with AuNPs, following pretreatment with ferrostatin-1 or erastin for 2 h at 30°C. After incubation and 5 min centrifugation, pellets were suspended with PBS and stained with DHE or HPF. After dyeing, the fluorescence of each regent was analyzed using a FACSverse flow cytometer (Becton Dickinson, USA).

### Lipid Peroxidation Assay

To monitor membrane lipid peroxidation, malondialdehyde (MDA) levels, an oxidative lipid marker, were measured [53]. First, the cells were incubated with AuNPs, AuNPs pretreated with ferrostatin-1, or erastin for 2 h at 30°C. Following incubation, cells were centrifuged, and pellets were then blended with lysis buffer (2% Triton-X 100, 1% SDS, 10 mM Tris-HCl, 1 mM EDTA, and 100 mM NaCl, pH 8.0). Then, cells were sonicated on ice using an ultrasonic sonicator (Sonics, USA) followed by centrifugation. Next, the supernatant was blended with thiobarbituric acid in 5% trichloroacetic acid. The mixture was warmed at 95°C for 30 min and then cooled on ice. The absorbance of each sample was estimated at 532 nm. Finally, MDA levels were assessed based on standard curves (created based on the manufacturer’s suggestions, Sigma).

### Evaluation of Membrane Integrity

Cellular membrane integrity is an indispensable condition for cell survival [54]. Propidium iodide (PI, Sigma) is a red-fluorescent stain that cannot pass through the membrane of living cells [55]. Due to its impermeable property, it is used to distinguish between healthy and necrotic cells [56]. Additionally, PI is reported to stain damaged membranes in both eukaryotes and prokaryotes [57]. Therefore, the PI assay was implemented to identify membrane integrity loss. First, we dissolved the PI in H_2_O. Next, cells were treated with AuNPs, AuNPs pretreated with ferrostatin-1, or erastin at 30°C. After 2 h incubation, cells were centrifuged at 12,000 rpm for 5 min, and then pellets were mixed with PBS. Finally, cells were stained with 10 μg/ml of PI, and fluorescence intensity of PI was detected using Eclipse Ti-s microscope (Nikon, Japan).

### Evaluation of Oxidative DNA Damage

8-hydroxydeoxyguanosine (8-OHdG) is a dominant derivative produced by DNA oxidation. Thus, it is used as an indicator to determine oxidative DNA damage [58]. The 8-OHdG levels were quantified by the competitive enzyme immunoassay method in our paper, using an Oxiselect Oxidative DNA Damage ELISA Kit (Cell Biolabs Inc., USA), according to the manufacturer’s instructions. The cells were incubated for 2 h at 30°C with AuNPs, AuNPs pretreated with ferrostatin-1, or erastin. As described in our previous study, DNA was extracted in an extraction solution (1% SDS, 2% Triton X-100, 100 mM NaCl, 10 mM Tris-Cl , 1 mM EDTA, and 10 mg/ml proteinase K) [59]. The absorbance of 8-OHdG was evaluated at 450 nm using an ELISA microplate reader.

### Evaluation of Mitochondrial Membrane Damage

In our investigation, 3,3'-dihexyloxacarbocyanine iodide (DioC_6_(3), Invitrogen, USA) and MitoTracker Green (Invitrogen) were used to observe mitochondrial damage [60]. Both are mitochondrial-selective labels and green fluorescence. The cells were incubated for 2 h at 30°C with AuNPs, AuNPs pretreated with ferrostatin-1, or erastin. Following incubation, the cells were centrifuged for 5 min at 12,000 rpm, and the supernatant was eliminated. Then, 1 ml PBS was mixed with the pellets. The samples were treated with 1 μM DioC_6_(3) to confirm mitochondrial membrane hyperpolarization, and 100 nM MitoTracker Green were treated on samples to monitor mitochondrial membrane density. After dyeing, DioC_6_(3)-stained samples and MitoTracker Green-stained samples were incubated for 30 min, and then fluorescence was determined using a microscope.

### Detection of other DNA Damage

To assess chromatin condensation, 4',6-diamidino-2-phenylindole (DAPI, Sigma) was used. *S. cerevisiae* cells were treated with AuNPs, AuNPs pretreated with ferrostatin-1, or erastin and incubated for 2 h at 30°C. After centrifugation, the cell pellets were resuspended in 1 mL of PBS, stained with 1 μg/ml DAPI, and incubated for 20 min in the dark. DNA fragmentation was analyzed using the terminal deoxynucleotidyl transferase dUTP nick end labeling (TUNEL) assay with an In Situ Cell Death Detection Kit (Roche Applied Science, Switzerland). For this assay, cells were treated with AuNPs, AuNPs pretreated with ferrostatin-1, or erastin and incubated for 2 h at 30°C. The cells were then washed with PBS and fixed with 2% paraformaldehyde on ice for 1 h. Subsequently, the fixed cells were permeabilized using a solution containing 0.1% Triton X-100 and 0.1% sodium citrate on ice for 2 min, followed by incubation with the TUNEL reaction mixture for 1 h at 37°C. Fluorescence intensity for each sample was measured using a FACSVerse flow cytometer.

### Detection of Metacaspases Activity

The MCA1/YCA1 gene has been recognized for coding for a metacaspase concerned in cell death of *S. cerevisiae* [61, 62]. To assess whether AuNPs trigger metacaspase activation, the CaspACE FITC-VAD-FMK In Situ Marker (Promega, USA) was employed. FITC-VAD-FMK is cell permeable and irreversibly labels activated caspases in apoptotic cells [63, 64]. Briefly, harvested cells were incubated with AuNPs, AuNPs pretreated with ferrotstatin-1 or erastin for 2h at 30°C. Next, incubated cells were centrifuged at 12,000 rpm for 5 min. And then, cells suspended with 1 ml PBS were stained with 5μM FITC-VAD-FMK (dissolved in DMSO) for 30min at 37°C. Finally, the intensity of CaspACE FITC-VAD-FMK was estimated using a FACSVerse flow cytometer.

### Examination of Ferroptosis-Like Response

N-(4-Diphenylphosphinophenyl)-N'-(3,6,9,12-tetraoxatridecyl)perylene-3,4,9,10-tetracarboxydiimide (Liperfluo, Japan) is a probe that reacts selectively with lipid peroxides [65, 66]. Therefore, it is suitable for observing ferroptotic responses due to lipid peroxidation. Before the full-scale experiment, Liperfluo was dissolved in DMSO. First, harvested cells were incubated with AuNPs, AuNPs pretreated with ferrotstatin-1 or erastin for 2 h at 30°C. After incubation, the cells were centrifuged at 12,000 for 5 min and washed with 100 μl PBS. Next, the cells were stained with 1 μM Liperfluo and incubated for 30 min at 37°C. Following incubation, the cells were washed twice with 100 μl PBS. Finally, fluorescence intensity was analyzed using a spectrofluorophotometer at wavelengths of 488 nm (excitation) and 550 nm (emission).

### Statistical Analysis

All experiments were performed in triplicate, with results presented as means ± standard deviation. The normality of data distribution was assessed using the Shapiro-Wilk test. Statistical analyses were conducted using analysis of variance (ANOVA) followed by Tukey’s post-hoc test for comparisons among three groups. These analyses were performed with SPSS software (version 25, SPSS/IBM, USA). Differences between groups were considered statistically significant when *p* < 0.05.

## Results and Discussion

Ferroptosis represents a unique form of programmed necrosis, characterized by mechanisms distinct from traditional cell death pathways, and holds promise as a potential approach for immunotherapy and cancer treatment [67, 68]. This type of cell death has been identified not only in mammals like humans and mice but also in organisms such as yeast and bacteria [69]. However, there are notable limitations when studying ferroptosis in eukaryotic microorganisms and prokaryotes. Although yeast and bacteria contain ferroptosis-associated proteins, genes, and enzymes, many of their roles remain poorly understood or unidentified. While ferroptosis itself is not directly observed in *S. cerevisiae*, which serves as the model organism in our study, the presence of glutathione (GSH) and GPx4-like protein (GPx3) has been confirmed, and lipid peroxidation has also been reported in this species [43, 70, 71]. Based on these findings, our research focuses on investigating ferroptosis-like cell death in *S. cerevisiae*.

### Induction of Cell Death in *S. cerevisiae*

**Fig. 1:**
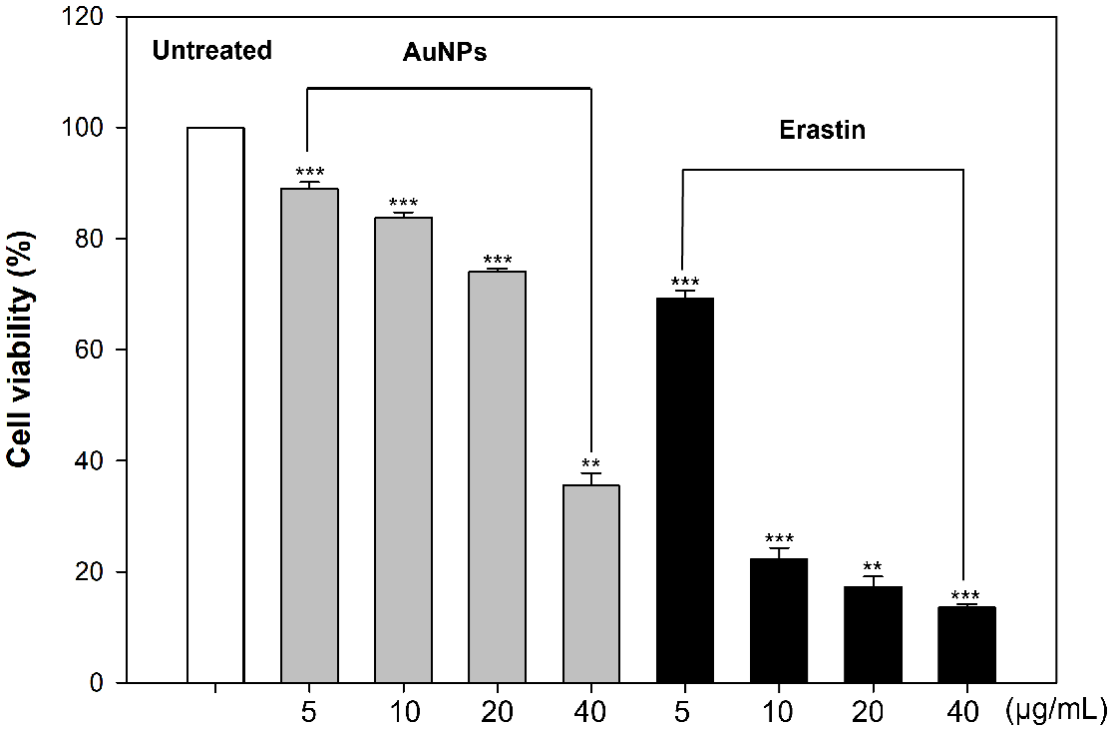
Cell viability of *S. cerevisiae* after treatment with AuNPs and erastin was measured by 3-(4,5-dimethylthiazol- 2-yl)-2,5-diphenyltetrazolium bromide (MTT) assay. Experiments were conducted in triplicate independently, and the results represent the average, standard deviation, and p values from three experiments (*p < 0.1; **p < 0.05; ***p < 0.01 vs. untreated sample).

AuNPs have long been applied to biotechnology based on their conjugation with various biomolecules. Furthermore, AuNPs targeting cell membrane penetration or drug delivery have been researched and developed for nanomedical applications. In cancer cells, AuNPs interact with cell cycle-related proteins, inhibiting cell cycle and proliferation [72]. Due to the size, shape, and surface-dependent properties of AuNPs, they act as cell death regulators, resulting in cell death, such as apoptosis, necrosis, or autophagy. Moreover, AuNPs and erastin are well-established inducers of cell death in multiple biological systems, including mammals, bacteria, and fungi. [12, 73]. Particularly, erastin used as a positive control in our experiments is a representative ferroptosis activator [74]. Although there is still a lack of ferroptosis-related research in eukaryotic microorganisms; *S. cerevisiae* is considered a typical yeast for ferroptosis [69]. Therefore, to determine the concentration at which necrotic death begins within *S. cerevisiae*, an MTT assay was conducted to examine cell viability. AuNPs were treated in cells by 5, 10, 20, and 40 μg/ml and erastin by 5, 10, 20, 40 μg/ml. As a result, cell viability decreased dose-dependently, and absorbance decreased rapidly in 40 μg/ml AuNPs and 10 μg/ml erastin (Fig. 1). Thus, this data suggested that cell death can initiate at 40 μg/ml AuNPs and 10 μg/ml erastin. Further experiments were conducted to identify the features that helped launch the ferroptotic cell death based on these concentrations.

### Overloading of Intracellular Iron

**Fig. 2:**
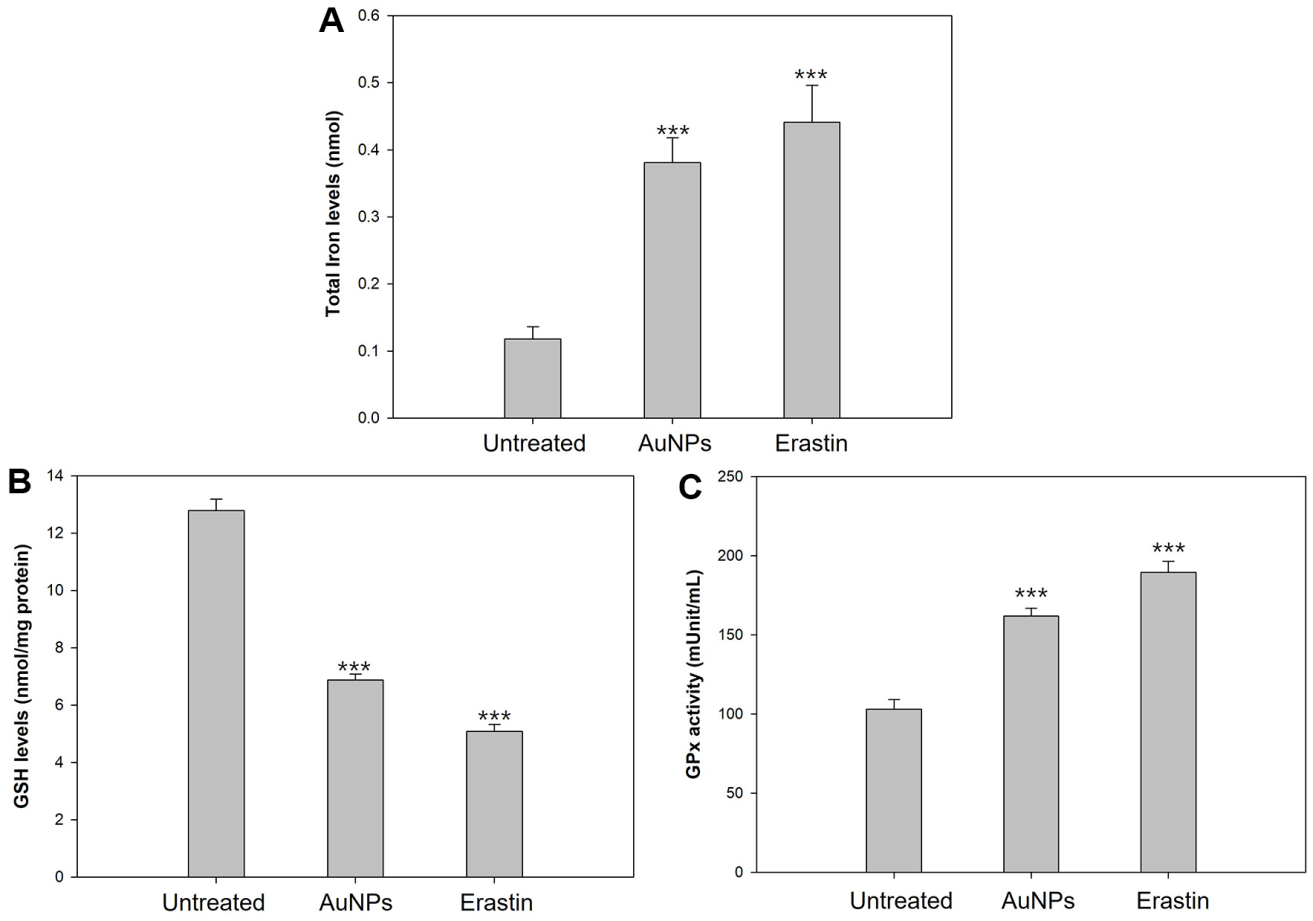
Identification of prerequisites for ferroptosis to execute. (**A**) Intracellular iron accumulation. (**B**) Intracellular glutathione (**GSH**) levels. (**C**) Glutathione peroxidase (GPx) activity. Experiments were conducted in triplicate independently, and the results represent the average, standard deviation, and p values from three experiments (*p < 0.1; **p < 0.05; ***p < 0.01 vs. untreated sample).

Iron is a vital element necessary for various biological processes, including respiration, cellular metabolism, DNA synthesis, and repair [75]. The regulation of iron levels is tightly controlled through coordinated mechanisms; however, an imbalance in iron homeostasis can lead to cellular damage and the generation of free oxygen radicals [76]. For instance, disruption of iron regulation is a critical factor that facilitates the onset of ferroptosis [77]. Despite this, the exact mechanisms by which iron deposition triggers ferroptosis remain unclear. However, several papers have demonstrated that overload of intracellular iron leads to the initiation of ferroptosis-like death, based on evidence that ferroptosis is inhibited by iron-binding complexes (deferoxamine or ciclopirox) [28, 51, 78]. To address this, we examined whether AuNPs contribute to iron overload in *S. cerevisiae* compared to erastin, a known inducer of intracellular iron accumulation. As shown in Fig. 2A, 40 μg/ml AuNPs had more than twice the total iron levels compared with the untreated cells, which showed levels that were close to 10 μg/ml erastin. The results showed that cells exposed to AuNPs exhibited elevated total iron levels, comparable to those observed in cells treated with erastin. This suggests that AuNPs promote iron accumulation in *S. cerevisiae*, potentially leading to ferroptosis.

### Depletion of Intracellular GSH and GPx Inactivation

A key factor contributing to ferroptosis is the depletion of glutathione (GSH) and the inactivation of glutathione peroxidase (GPx), both of which result from cysteine (Cys) deficiency [79]. GSH plays a crucial role in the cellular antioxidant system, and insufficient Cys disrupts its synthesis. When intracellular GSH levels drop below approximately 10%, the antioxidant defense system becomes compromised. Additionally, a lack of Cys leads to GPx inactivation, a critical inhibitor of ferroptosis. Given that *S. cerevisiae* possesses GPx, which functions similarly to GPx4, we assessed intracellular GSH levels and GPx activity to evaluate the impact of AuNPs on ferroptosis [80]. Our findings revealed that AuNP-treated cells exhibited significant reductions in both GSH levels and GPx activity, suggesting that AuNPs induce pro-ferroptotic conditions in *S. cerevisiae* through Cys deficiency Most ferroptosis inducers interfere with Cys import, hindering GSH synthesis [81, 82]. Erastin, used as a positive control, depletes Cys while blocking Xc^-^-mediated import [83]. To determine whether AuNPs similarly impair GSH synthesis due to Cys deficiency, we analyzed GSH levels in AuNP-treated cells and observed reductions comparable to those in erastin-treated cells (Fig. 2B). Furthermore, intracellular Cys depletion not only weakens the antioxidant defense system but also inhibits GPx protein expression [84]. To assess GPx activity, we measured the oxidation rate from NADPH to NADP^+^, revealing a threefold decrease in the NADP^+^/NADPH ratio in AuNP-treated cells compared with untreated cells (Fig. 2C) [85]. These results indicate that AuNPs lead to Cys deficiency, thereby inhibiting GSH synthesis and GPx activity, ultimately promoting ferroptotic conditions in *S. cerevisiae*.

### Accumulation of Lipid ROS

**Fig. 3:**
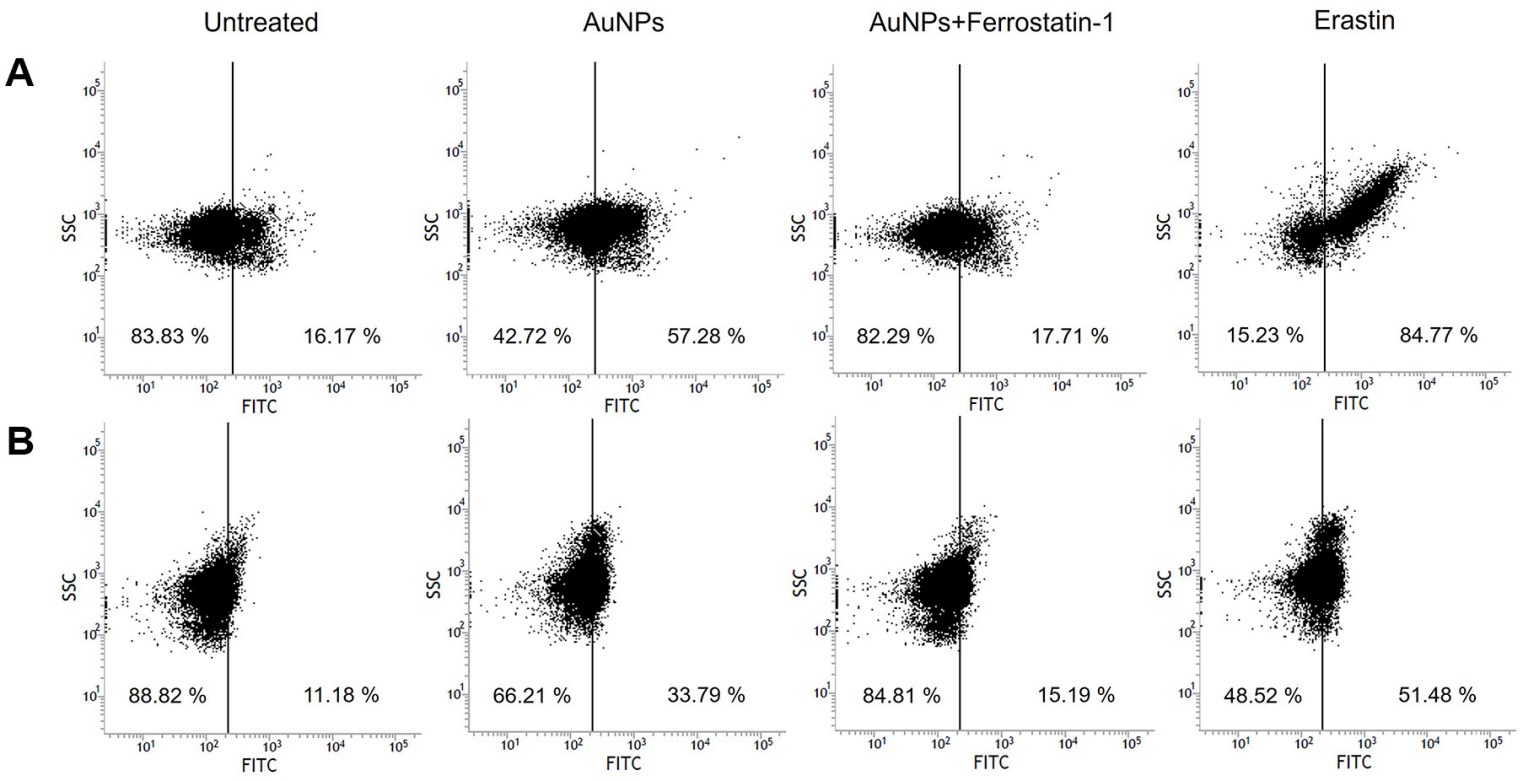
Flow cytometry analysis of intracellular reactive oxygen species (ROS) generation. (**A**) Superoxide and hydrogen peroxide generation was detected using the dihydroethidium (DHE) assay. (**B**) Hydroxyl radical generation was detected using the 3'-(p-hydroxyphenyl) (HPF) assay.

The accumulation of lipid ROS is the direct trigger of ferroptosis, whereas iron overload, GSH depletion, and GPx inactivation contribute to its progression. Iron accumulation leads to the production of highly reactive hydroxyl radicals (OH^-^) via the Fenton reaction, while cysteine or GSH deficiency exacerbates oxidative stress by impairing the cellular antioxidant defense system [86]. Similarly, cysteine or GSH deficiency exacerbates oxidative stress by impairing the cellular antioxidant defense system. Given that ferroptosis is an iron-dependent oxidative cell death process, it is closely linked to ROS production. Many studies have reported that AuNPs mediate high levels of ROS generation [87-89]. Furthermore, ferroptosis is also considered a ROS-reliant response because it is an iron-dependent oxidative cell death [90, 91]. To determine whether AuNPs trigger intracellular ROS accumulation, we used dihydroethidium (DHE) to detect superoxide (O_2_^-^) and hydrogen peroxide (H_2_O_2_), and hydroxyphenyl fluorescein (HPF) to detect OH^-^. Our results showed that cells treated with AuNPs or erastin exhibited significantly higher DHE and HPF fluorescence intensity compared with untreated cells (Fig. 3), indicating increased intracellular ROS levels. Furthermore, pretreatment with ferrostatin-1, a lipid ROS scavenger, reduced this fluorescence intensity in AuNP-treated cells, confirming that the ROS generated by AuNPs include lipid ROS. These findings demonstrate that AuNP-induced iron overload and cysteine deficiency contribute to the generation of toxic lipid ROS, ultimately driving ferroptotic cell death in *S. cerevisiae*.

### Occurrence of Lipid Peroxidation

**Fig. 4:**
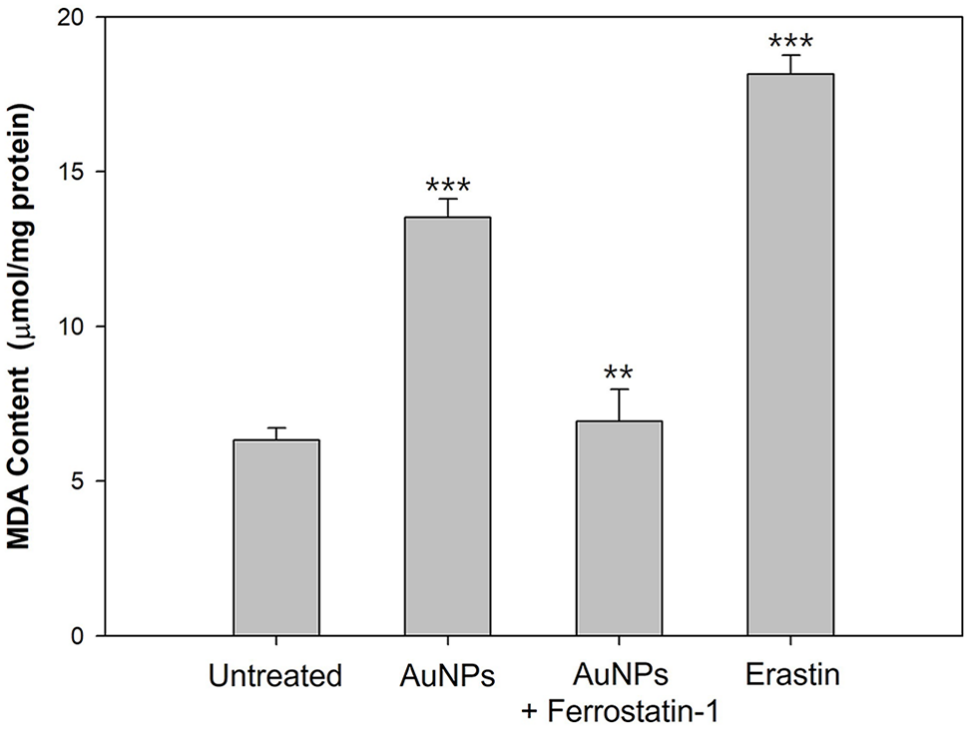
Lipid peroxidation was measured by the TBARS assay in *S. cerevisiae*, and an increase in malonaldehyde (MDA) levels indicates the peroxidation of lipids. Experiments were conducted in triplicate independently, and the results represent the average, standard deviation, and p values from three experiments (*p < 0.1; **p < 0.05; ***p < 0.01 vs. untreated sample).

Lipid peroxidation during ferroptosis is closely linked to iron metabolism and the activity of GPx enzymes. Under normal conditions, GPx4 inhibits lipid peroxidation through the cellular antioxidant system. However, when GPx is inactivated, oxidative damage to plasma membrane lipids occurs, ultimately triggering ferroptosis. Additionally, reactive oxygen species (ROS) generated from iron accumulation interact with polyunsaturated fatty acids (PUFAs), further driving lipid peroxidation. Due to intracellular ROS accumulation and GPx inhibition, cells lose their ability to eliminate lipid peroxides, leading to ferroptosis initiation [69, 92]. To verify whether AuNP-induced ROS accumulation and GPx loss promote lipid peroxidation, we measured malondialdehyde (MDA), a terminal product of lipid peroxidation [93]. Our results showed significantly higher MDA levels in AuNP- or erastin-treated cells compared with untreated cells (Fig. 4), indicating increased membrane lipid peroxidation. Conversely, pretreatment with ferrostatin-1, a lipid ROS scavenger, reduced MDA levels to those observed in untreated cells. These findings demonstrate that AuNP-induced GPx inactivation and iron overload contribute to lipid ROS generation and subsequent membrane lipid peroxidation, ultimately driving ferroptotic cell death in *S. cerevisiae*.

### Rupture of Plasma Membrane

**Fig. 5:**
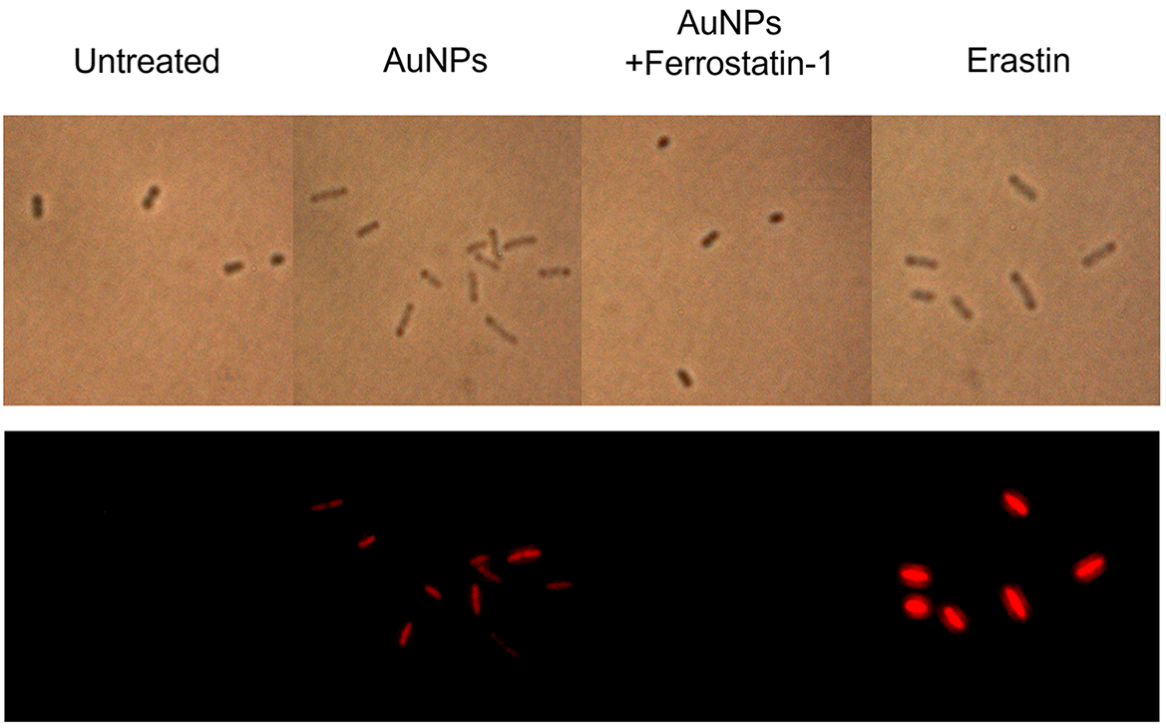
Membrane damage was detected by propidium iodide (PI) staining in *S. cerevisiae*.

In most organisms, the plasma membrane consists of a phospholipid bilayer, making it particularly vulnerable to lipid peroxidation caused by oxidative stress. Oxidized lipids reduce membrane thickness, disrupt the activity of membrane-associated enzymes, and compromise structural integrity [94, 95]. These changes impair essential processes such as diffusion and ion transport, ultimately leading to membrane permeability loss. During ferroptosis, lipid peroxidation induces membrane rupture rather than the formation of blisters [96, 97]. However, the precise mechanism by which lipid peroxidation leads to membrane rupture remains unclear. To assess membrane integrity, we measured propidium iodide (PI) fluorescence, which indicates the extent of membrane rupture. Our results showed that AuNP-treated cells exhibited significantly higher red fluorescence, similar to erastin-treated cells (Fig. 5), confirming membrane damage. In contrast, pretreatment with ferrostatin-1 prevented this effect, as fluorescence levels remained comparable to those of untreated cells. These findings demonstrate that AuNP-induced lipid peroxidation leads to membrane rupture, strongly suggesting that these features contribute to ferroptotic cell death in *S. cerevisiae*. Our results showed that AuNP treatment significantly compromised membrane integrity, while ferrostatin-1 mitigated this effect. These findings confirm that lipid peroxidation contributes to cellular membrane damage in *S. cerevisiae*.

### Monitoring of Oxidative DNA Damage

**Fig. 6:**
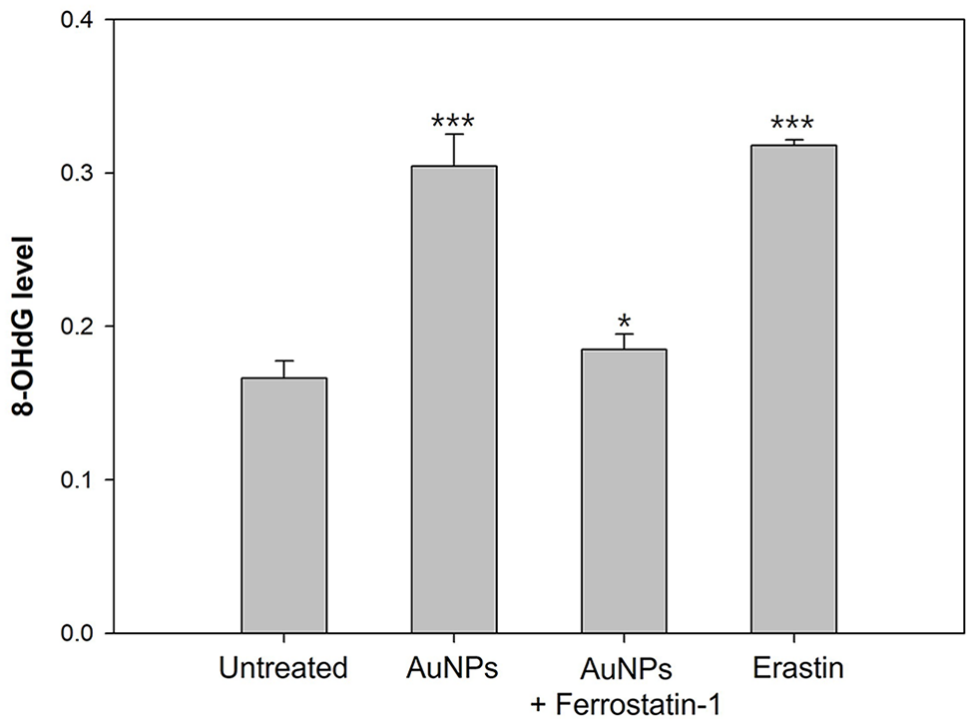
Oxidative DNA damage was performed by 8-OHdG quantitation in *S. cerevisiae*. Experiments were conducted in triplicate independently, and the results represent the average, standard deviation, and *p* values from three experiments (*p < 0.1; **p < 0.05; ***p < 0.01 vs. untreated sample).

Oxidative stress generated by ROS targets DNA strands and bases, compromising genome stability during processes such as DNA replication and repair. This genotoxic stress leads to DNA damage and increases the risk of mutations. Guanine, a purine base that pairs with cytosine, is particularly susceptible to oxidation, forming 8-hydroxy-2'-deoxyguanosine (8-OHdG), a well-known marker of oxidative DNA damage. These DNA adducts interfere with DNA polymerase activity, preventing the proper recognition of guanine and causing mutations. In yeast, cysteine residues are recognized to act as sensors to counter oxidative responses [98, 99]. Moreover, oxidative stress promoted by cysteine depletion impact on biomolecules negatively [100]. Under endogenous oxidative stress, the modification of the DNA base is induced, and guanine is oxidized at this time, forming 8-OHdG as an oxidative derivative. Therefore, the degree to which AuNP-induced ROS impairs DNA was quantified through intracellular 8-OHdG levels. The results showed a significant increase in 8-OHdG levels in AuNP-treated cells compared with untreated cells (Fig. 6), indicating AuNP-induced oxidative DNA damage. In contrast, pretreatment with ferrostatin-1 reduced 8-OHdG levels, suggesting that AuNP-generated ROS plays a direct role in DNA oxidation. Furthermore, considering the previously observed iron overload and GSH depletion in AuNP-treated cells, our findings suggest that AuNP-induced oxidative stress causes severe damage to both membrane lipids and DNA, contributing to ferroptotic cell death in *S. cerevisiae*.

### Confirmation of Mitochondrial Membrane Damage

**Fig. 7:**
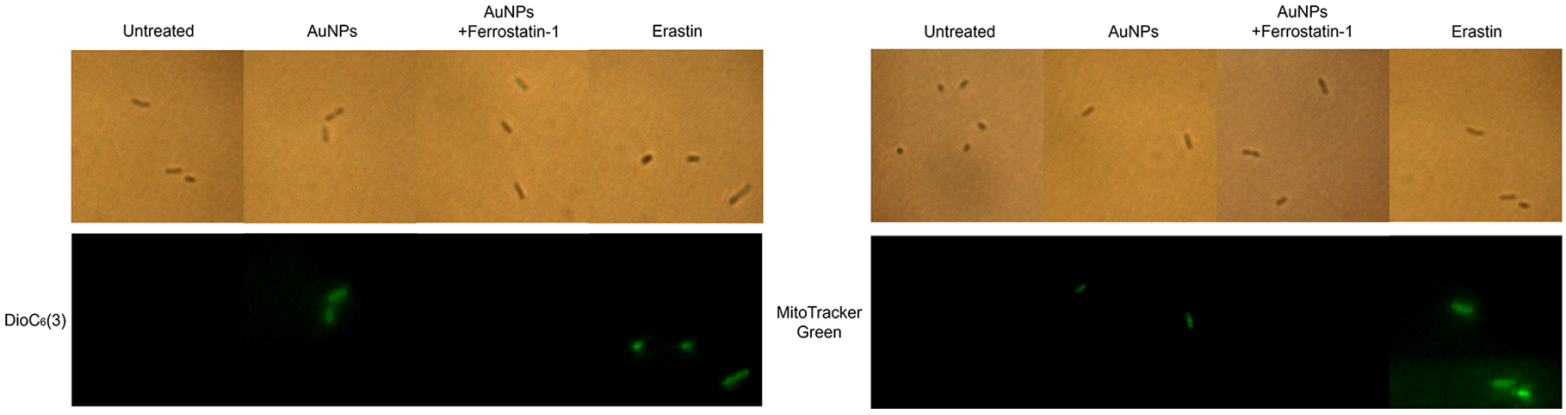
Mitochondrial membrane potential hyperpolarization was detected using DioC_6_(3), and mitochondrial membrane density was detected using MitoTracker Green.

The previously observed iron overload and cysteine deprivation also directly damage the mitochondrial membrane [101, 102]. Additionally, cysteine deprivation is involved in the electron transport chain activity, which induces mitochondrial membrane potential (MMP) hyperpolarization [103, 104]. MMP hyperpolarization has also been reported to increase mitochondrial membrane density. Therefore, using DioC_6_(3) and MitoTracker Green, MMP hyperpolarization and mitochondrial mass were assessed, respectively. Consequently, both experiments identified that AuNPs-treated cells exhibited green fluorescence similar to those treated with erastin (Fig. 7). Furthermore, pretreatment of ferrostatin-1 in AuNPs-treated cells weakened these effects. These outcomes implied that AuNPs-induced iron overload and cysteine deficiency result in mitochondrial membrane damage.

### No appearance of Apoptotic Hallmarks and Verification of Ferroptosis-Like Death

**Fig. 8:**
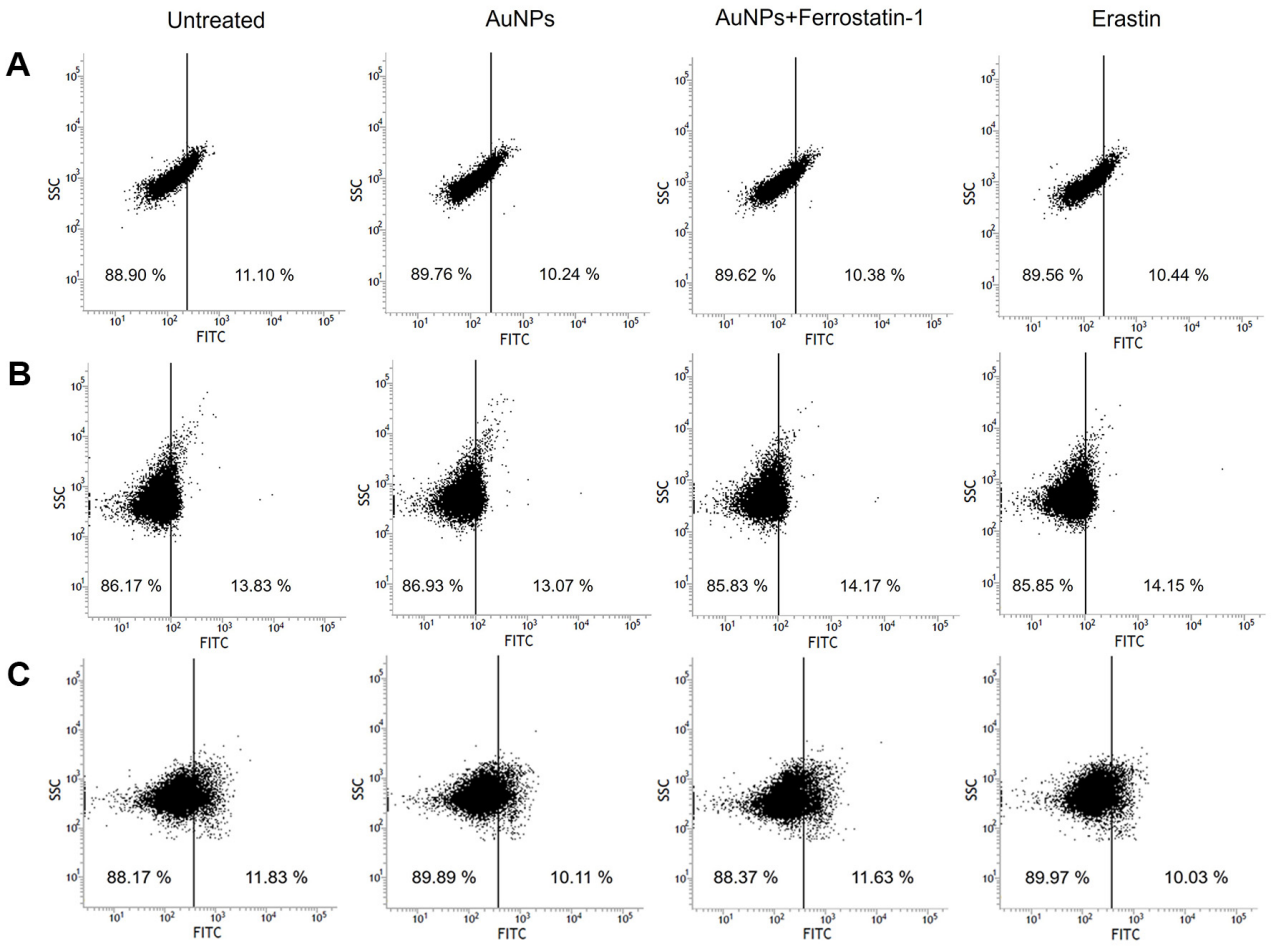
DNA damage and metascaspase activation was measured in *S. cerevisiae*. (**A**) Chromosomal concentration. (**B**) DNA fragmentation. (**C**) Flow cytometric analysis of by CaspACE FITC-VAD-FMK in *S. cerevisiae*.

**Fig. 9:**
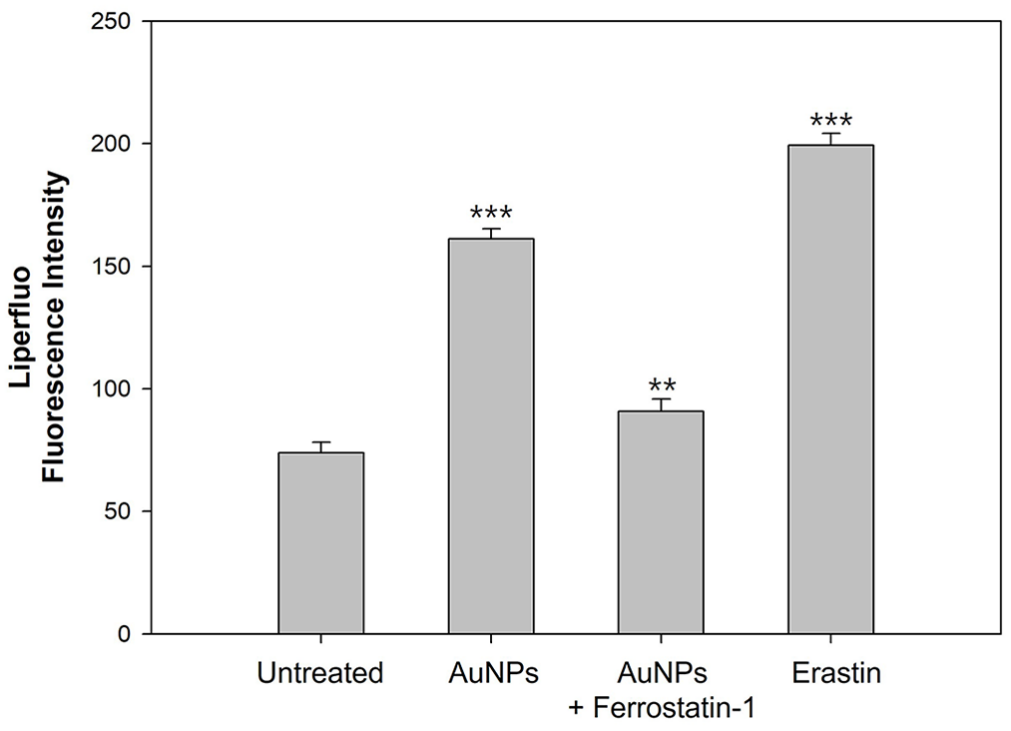
Ferroptotic cells were detected by the Liperfluo in *S. cerevisiae*. Experiments were conducted in triplicate independently, and the results represent the average, standard deviation, and p values from three experiments (*p < 0.1; **p < 0.05; ***p < 0.01 vs. untreated sample).

In ferroptosis, there are no morphological or biochemical changes similar to apoptosis [19, 83]. Therefore, our investigations were performed to detect whether AuNPs treatment represents chromatin condensation, DNA fragmentation, and caspase activity, which are standard hallmarks of apoptosis [105-107]. As shown in Fig. 8A and 8B, AuNPs treatment did not represent chromatin condensation and DNA fragmentation in *S. cerevisiae*, and it was a little different from the pretreatment of ferrostatin-1 in AuNPs-treated cells. Likewise, in Fig. 8C, there was little difference in metacaspases activity in all samples. Thus, these data indicated that AuNPs have no characteristics of apoptosis, suggesting that 40 μg/ml AuNPs are more suitable for inducing ferroptosis in *S. cerevisiae* than apoptosis. Ferroptotic cell death is driven by overpowering lipid peroxidation [25, 92]. We previously observed massive lipid peroxidase due to AuNPs and employed Liperfluo to identify indirectly whether ferroptotic cells appears. As shown in Fig. 9, the cells treated with AuNPs showed more than twice the fluorescence intensity compared to the untreated cells. On the other hand, pretreatment of ferrostatin-1 in AuNPs-treated cells exhibited reduced fluorescence. This result suggests that ferroptotic cells appear in *S. cerevisiae* due to lipid peroxidation caused by AuNPs. Therefore, our experiments provided the potential for AuNPs to become a ferroptosis inducer.

## Conclusion

Ferroptosis, as a form of non-apoptotic cell death, exhibits distinct characteristics that differentiate it from apoptosis. Under oxidative stress, DNA damage in apoptotic cells is typically accompanied by hallmark features such as chromatin condensation and DNA cleavage, whereas these features are absent in ferroptosis. Additionally, caspase activation, a key process in apoptosis, does not occur during ferroptosis. Consistent with this, our study did not observe chromatin condensation, DNA fragmentation, or meta caspase activation, which are associated with apoptosis. Although several hypotheses exist to explain the differences between ferroptosis and apoptosis, the exact mechanisms underlying these distinctions remain unclear. Furthermore, the lack of specific markers for ferroptosis has made it more challenging to study compared to apoptosis. Despite these obstacles, ongoing research into ferroptosis holds significant promise for developing treatments for a variety of diseases.
